# From action to ethics: A process-relational approach to prosocial development

**DOI:** 10.3389/fpsyg.2023.1059646

**Published:** 2023-02-14

**Authors:** Jeremy I. M. Carpendale, Beau Wallbridge

**Affiliations:** Department of Psychology, Simon Fraser University, Burnaby, BC, Canada

**Keywords:** moral development, prosocial, infancy, socio-moral development, emotional involvement, process-relational approach, engagment, worldviews

## Abstract

Explaining how children first become active prosocial and then later moral agents requires, we argue, beginning with action and interaction with others. We take a process-relational perspective and draw on developmental systems theory in arguing that infants cannot be born knowing about prosociality or morality or anything else. Instead, they are born with emerging abilities to act and react. Their biological embodiment links them to their environment and creates the social environment in which they develop. A clear distinction between biological and social levels cannot be made in the context of ongoing development because they are thoroughly interwoven in a bidirectional system in which they mutually create each other. We focus on infants’ emerging ability to interact and develop within a human developmental system, and prosociality and morality emerge at the level of interaction. Caring is a constitutive aspect of the forms of experience in which infants are embedded in the process of becoming persons. Infants are immersed in a world of mutual responsiveness within caring relationships that are infused with concern, interest, and enjoyment. In such a developmental system, infants become persons when they are treated as persons.

## Introduction

Our relations with others, from interpersonal caring to social justice, are central in human cultures. Thus, it is crucial to account for the emergence of these skills in human development. We argue that addressing the question of how children become active prosocial agents requires beginning with action and interaction with others. By contrast, one current claim is that a moral sense is innate. We have argued that such claims are problematic at the conceptual, methodological, and biological levels (e.g., Carpendale et al., 2021a). Another claim is that socio-moral development can be explained in terms of children coming to accept the behavioral norms of their culture. Although this aspect of development needs to be explained, such conformity to rules is not a complete account of morality because as well as accepting some norms, children and adolescents also challenge and change societal norms, and this needs to be explained in a full developmental account (e.g., Carpendale et al., 2013).

We argue that infants cannot be born knowing about morality or anything else. Instead, they are agents, born after 9 months of development, with emerging abilities to interact, act, and react. Their biological embodiment links them to their environment, and it also creates the social environment in which they develop. For example, human infants’ helplessness requires that they be cared for, and this results in a social–emotional context for their development (e.g., Portmann, 1990; Savage-Rumbaugh and Fields, 2011). Infants’ neurological development is shaped by this social experience (e.g., Carpendale and Lewis, 2021). This is a developmental system account in which a clear distinction between biological and social levels cannot be made in the context of ongoing development because they are thoroughly interwoven in a bidirectional system in which they mutually create each other. In our account, we focus on infants’ emerging ability to interact and develop within a human developmental system. Prosociality and morality emerge at the level of interaction. We argue that the concern for others that underlies moral development does not need to be added later to human relations, but rather that caring is a constitutive aspect of the forms of experience in which infants are embedded and require in the process of becoming persons. The world in which infants are immersed from the beginning is saturated with engagement. They live in a world of mutual responsiveness, within caring relationships that are infused with concern, interest, and enjoyment. In such a developmental system, infants become persons when they are treated as persons (Carpendale and Lewis, 2021).

To understand and evaluate research on prosocial development, it is important to be clear that the presuppositions researchers start from matter for the theories they work with because “starting points have a tendency to haunt us all the way through to our theoretical conclusions” (Jopling, 1993, p. 290). We compare two starting points in terms of the presuppositions regarding cognition and relations on which theories are based: (1) either cognition is assumed to be necessary to make interpersonal relations possible or (2) it is relations that are primary, and cognition emerges from interaction within such relations (Jopling, 1993). The first approach is the cognitivist perspective, also referred to as a Cartesian, split, mechanistic worldview (Overton, 2015). The second approach begins with relations as primary and as the explanation for the development of cognition. From this perspective, “gesture and communication come before mind and self” (Jopling, 1993, p. 526). Relations are primary and they make higher-order cognitive relations possible, not the other way around (Jopling, 1993). These two approaches to understanding prosocial and moral relations start from opposite directions. One is exemplified by the subtitle for Pinker (1994) book, “*The Language Instinct*,” that is, “*How the mind creates language*.” This begins from, but does not explain, the mind. This framework contrasts with the alternative that concerns how communication, thinking, and mind emerge from interaction (e.g., Mead, 1934). We argue that cognitivism presupposes what it should explain. Instead, we begin with process, relations, systems, and interaction.

Although we have mentioned both prosocial action and morality, in this article, we focus on the development of prosocial action. But this development is linked to morality, and it is important to think about this distinction. We discuss this in the following section. Then, we briefly and critically examine nativist approaches to this area of development and claims of innate infant morality. We then introduce a process-relational, action-based approach to development, and in the next section apply this perspective to understanding the development of prosocial action. Following this, we consider different forms of prosocial action, such as helping, sharing, and comforting, and how they might initially develop somewhat independently. Finally, we consider the methodological implications that follow from the process-relational approach.

## Prosocial action, morality, and interaction

There is an important distinction between prosocial action and morality, but we suggest that to understand development in these areas, we should resist assuming a hard dividing line between them. Prosocial action benefits others. But so does moral action, so they overlap in this way. Morality is a broader domain than prosociality, as it includes prosocial action and also concerns obligations and moral norms regarding what is right and wrong. This then raises the question of where moral norms concerning right and wrong come from. How do they develop? Explaining the origin of such moral norms is a difficult but important task that we address elsewhere (Carpendale et al., 2013, 2021a). We explicate the problem of accounting for moral norms and critique the following two commonly proposed solutions: moral norms are either innate and explained in terms of biology, or moral norms are imposed on children through parents and culture. We critique claims that such norms could be innate or completely imposed on children and argue instead for an alternative that they emerge through interpersonal agreement. Since we take a developmental perspective on this issue and do not assume that moral norms pre-exist in either the individual’s biology or in the culture, we need to explain how they develop. From this perspective, it is important to think about forms of human interaction that gradually become more complex as morality emerges. When we look at interaction even in infancy, we see that the basic structure of communication involves responding to others; therefore, there is already something ethical in treating the other as a person. This is the structure of the interaction that infants experience and develop within (Jopling, 1993; Carpendale, 2018).

We argue that the skills we think of as linked to being a person emerge within relations, and these relations are fundamentally ethical because they are based on responsiveness, respect, and responsibility. Thus, these social relations in which persons develop are ethical in their foundations (Jopling, 1993). In addition, these interpersonal relations are mastered by children to become what we think of as individual cognitive skills, but they are social and ethical in their origins and their foundations. Thus, ethics is not something added later in development. It is already in some sense present within human relations. In this article, we focus on prosocial development and expand on early development from the perspective of an action-based or process-relational approach. To do so, we describe the emergence and development of infants’ enjoyment of interacting with others and their development of new ways to elicit such interaction.

We begin with intersubjectivity or lived activity (Jopling, 1993). By contrast, if researchers already start with individuals and cognitive skills, then it is necessary to figure out how to glue them back together, and reasons are needed for why they should care about each other (e.g., Dunfield, 2014; Tomasello, 2020). But if one begins from our starting point in relations, then caring is a foundational aspect of the structure of interaction that emerges at the beginning of parent–infant interaction. It is not something that has to be added later. Emotions, including interest and enjoyment, and curiosity are aspects of this interaction (Carpendale and Lewis, 2020, 2021).

## Nativism, infant morality, and process-relational approaches

Strong claims have been made regarding infants being born with innate morality (e.g., Hauser, 2006a,b; Hamlin et al., 2007; Mikhail, 2007; Bloom, 2010, 2012; Graham et al., 2013; Hamlin, 2013; Margolis and Laurence, 2013; Warneken, 2016). For instance, it has been claimed that infants “have a rudimentary moral sense from the very start of life” (Bloom, 2010, p. 46), that “genes (collectively) write the first draft [of the infant’s moral mind] into neural tissue” (Graham et al., 2013, p. 61), and that this first draft includes an abstract expectation of fairness (see Bian et al., 2018, p. 2705; Buyukozer Dawkins et al., 2019, p. 16).

Our developmental position contrasts with these current nativist claims regarding infants’ moral competence. Elsewhere, we and others have critiqued such claims from conceptual as well as methodological perspectives (e.g., Carpendale et al., 2021b,c; Carpendale and Lewis, 2021). Here, we briefly reiterate that although these claims of innate knowledge might seem to be based on biology, these researchers do not cite current research in biology and instead what is defining about their claims is the notion of pre-existing moral knowledge or essentialism. These claims seem to clash with what is currently known about the functioning of genes (e.g., Fisher, 2006; Gottlieb, 2007; Meaney, 2010) as well as neural development (e.g., Stiles, 2009; Stiles et al., 2015). Thus, this is a curious nativism that seems to exist independently of biological knowledge, similar to Chomsky (2007) logical claims.

It might be thought that because we recognize the capacities that infants demonstrate at birth, we draw something from nativism, but this is not the case. Nativism presupposes pre-existence and essentialism (Lerner, 2016) rather than development. The assumption is that something must pre-exist. Instead, we draw on developmental systems theory according to which it is necessary to explain development, and we should do without the dichotomy between biology and social factors because infants’ biological characteristics structure their experience which, in turn, shapes infants’ biology in a bidirectional manner. Biological and social factors cannot be meaningfully separated, and the dichotomy is misleading because they mutually create each other (e.g., Griffiths and Stotz, 2000; Gottlieb, 2007). Our work is consistent with a systems biology that focuses on relations and the process of development rather than making claims of pre-existence and essentialism (Lerner, 2016). This developmental systems approach to biology that we draw on is consistent with neuroconstructivism, according to which neural pathways are shaped through experience (e.g., Mareschal et al., 2007; Stiles, 2009; Stiles et al., 2015).

Beyond the problematic biological assumptions on which nativism is based, we also criticize the assumptions regarding knowledge and thinking that nativism presupposes. Claims of innate knowledge rest on a representational view of knowledge and the view of thinking as computation, both assumptions that we and others have extensively critiqued (e.g., Heil, 1981; Carpendale et al., 2021a). By contrast, we argue that to understand human development, it is essential to begin with relations. Cognition cannot be prepared in advance of experience because it has to be meaningfully linked to the world. It develops in relations; it is not a mechanical connection.

## A process-relational, action-based worldview

We have argued that where we start from matters. Rather than starting with and thus presupposing individual cognition to explain relations, we begin instead with relations as primary. Many approaches presuppose humans as capable of living self-sufficient, isolated, independent lives. This presupposes a split between the first and third person; minds are presupposed as primary. According to this starting position, it is cognitive competence that makes relations possible. This requires what Hobson (2004) called a joining together account that presupposes a split between self and other. The problem with this starting assumption is that persons are presupposed yet that is what we must explain. By contrast, we take a differentiating out account according to which the development of individuals occurs as emerging out of relations. The relational view of persons is that they become persons in relation to others.

There are many sources of the broad worldview that we draw on. We draw on action-based approaches such as Mead (1934), Piaget (1965), Wittgenstein (2009), and Carpendale and Lewis (2021). These are currently referred to as process-relational approaches (Overton, 2015). They could be grouped into a family of approaches with interactivism (Bickhard, 2009; Allen and Bickhard, 2013), enactivism (e.g., Maturana and Varela, 1987; De Jaegher and Di Palolo, 2007; De Jaegher et al., 2010), and other approaches in cognitive science (e.g., Hutto and Myin, 2013). Jopling (1993) contrasts what he refers to as the philosophy of subjectivity which begins from cognition to explain relations with the philosophy of intersubjectivity or dialog, drawing on Buber and Levinas, which begins from relations. The focus on relations is also central in work in feminist theory and the idea of an ethic of care (e.g., Gilligan, 1982; Noddings, 1984). The foundational idea of human interdependence is clear in the African social and ethical concept of Ubuntu, that is, the idea that a person becomes a person in relation to other persons (Tutu, 1999; Waghid and Smeyer, 2012). This way of thinking about the importance of relations also seems to be embedded in Japanese and Chinese thinking (e.g., Carpendale and Lewis, 2021) and is central in many indigenous worldviews (e.g., Ross, 2006). Another source of approaches focused on relations derives from developmental systems theory in biology, in which this idea applies at all levels from genetics to neuroscience (Griffiths and Stotz, 2000; Gottlieb, 2007). These various approaches apply the idea of relations at contrasting levels from the cytoplasm and the synapse to society and social justice. It is a further task, however, to distinguish and analyze differences among these perspectives. We now consider approaches more well-known in psychology.

Within psychology, there are a variety of approaches taking what is currently referred to as 4E approaches to cognition, which acknowledge that human cognition is embodied, embedded, enacted, and extended (see the Oxford Handbook on 4E cognition; Newen et al., 2018). Although these approaches recognize that human cognition is embedded in relations, there is still considerable diversity and debate among researchers (Newen et al., 2018). To draw out the developmental implications of these approaches, we suggest that it is necessary to extend this way of thinking by adding an E for emergence (see Bickhard, 2009, on emergence). Furthermore, to get to the sixth E of ethics, we need to consider other Es. In particular, the E for emotions because our actions need to be linked meaningfully to the world. Emotions viewed as interpersonal relatedness and interest draw infants into relations with others (Hobson, 2012; Hammond and Drummond, 2019; Reddy and Vanello, 2022). Furthermore, another E should be added for enjoyment in interacting with others as a crucial ingredient in structuring the human developmental systems in which persons develop, and this is linked to interest (Jopling, 1993; Hammond and Drummond, 2019).

Within these interpersonal relations, we could also add the 4Rs related to these relations of response, responsibility, respect, and relational autonomy, as well as the 4Cs of care, compassion, concern, and connection. This brings out the ethical nature of such relations. The nature of the interaction is already ethical because we typically respond to others as persons, that is, as someone and not something (Spaemann, 2006), as in Buber (1958) distinction between human relations as “I-Thou” versus “I-it.” We argue that concern for others does not need to be added to human relations, but rather is a constitutive aspect of the forms of experience that infants are embedded in during the process of becoming a person.

Another approach that is similar to what we are proposing is currently referred to as a “second-person” approach (e.g., Reddy, 2018). This is presented as an alternative to approaches presupposing that development begins with the first-person perspective, referring to the subjective or experiencing perspective, and the third-person or the observer perspective. This sets up the traditional problem of other minds because the individual is assumed to have privileged access to her mind, the first-person perspective, and must make inferences about minds in other bodies, the third-person perspective. These assumptions presuppose the split between self and other and the problem of other minds. By contrast, the second-person perspective is the intersubjective, participant, or co-experiencing perspective (Fuchs, 2013). Second-person relations are fundamentally about direct engagement with, rather than observation of, the other (Fuchs, 2013; Reddy, 2018).

Engagement involves an immediate (i.e., unmediated and non-representational) way of interacting with others as whole persons—rather than as bodies, minds, or minds-within-bodies (Jopling, 1993; Fuchs, 2013). Second-person engagement is fundamentally characterized by addressing the other and being addressed in an act of “momentary openness to each other” (Reddy and Vanello, 2022, p. 254). These moments involve mutual responsiveness that gives spontaneity to the interaction, affording the possibility of creating novel shared experiences. Engagements do not exist within two first-person subjects, but rather *between* the interactants (Reddy, 2018).

As Reddy (2018) argues, this is the world of second-person engagement, and it is the world that infants are immersed in from the beginning. Thus, they are living in a world of addressing and being addressed, of mutual responsiveness, tied up in a caring relationship that is infused with strong emotions of concern, interest, and enjoyment. In such a developmental system, infants become persons when they are treated as persons (Carpendale and Lewis, 2020, 2021). It is through treating, or failing to treat, others as a person that infants learn the interpersonal consequences of their actions. From the current second-person perspective, this remains the core as children begin to develop an awareness of the perspectives involved in a moral conflict—including their own—which allows them to engage in a moral process of coordinating the conflicting perspectives (Mead, 1934; Piaget, 1965; Carpendale et al., 2013). Thus, morality does not need to be added to human relations, but rather is a constitutive aspect of the forms of experience that infants require to become a person.

We agree with the spirit of the second-person approach, but we question whether the term “second-person” is the best description of this intersubjective perspective. The term “second person” is in contrast to first-and third-person perspectives, but it is a bit odd because, at that point in early development, there is no perspective of a person, at least in the cognitive, mentalistic sense that is often assumed, instead, there are direct relations that are not mediated. Fuchs (2013) noted that he was aware that it is a problematic term but believed it is worth the cost.

## Accounting for prosocial development by beginning from relations

We take the perspective that the development of social knowledge emerges as social skills (Bibok et al., 2008). In understanding how young children enter the social, emotional, and moral forms of relations with others, we draw on the philosophy of intersubjectivity and dialog (Jopling, 1993) and the idea that “the reality of interpersonal relations is a given rather than a cognitive achievement” (Jopling, 1993, p. 292). This is a reversal of the cognitivist position that we critically evaluated earlier. We suggest that our perspective is Darwinian because we begin with relations. That is, we begin with something observable and explain instead of presupposing cognition. In discussing this approach, Jopling (1993) explicates ideas from Levinas and Buber according to which first-order ethical relations make higher-order cognitive relations possible, not the other way around. Caring is not something that is added later based on cognition. Instead, care and respect are part of the structure of the human developmental system that makes the development of cognition possible (Carpendale, 2020; Carpendale et al., 2021a).

In taking this perspective, the goal is to chart the gradual development within relations of young children’s social skills and emerging concern for others. In the initial research on the topic of young children’s helping, Rheingold (1982, p. 115) suggested that a “fundamental characteristic of human infants that underlies helping, and many other prosocial behaviors, is their interest in people and their activities.” This insight was overlooked in Warneken (2016) claims that there are biological foundations for infants’ helping and that social experience is of little importance in its emergence. In evaluating this claim, it is essential to clarify what is meant by biological foundations because, in one sense, everything has biological foundations. But Warneken (2016) means that prosociality is primarily explained by biology, that it pre-exists in biology. This claim, however, appears to be inconsistent with current biology and an understanding of the functioning of genes (e.g., Gottlieb, 2007; Meaney, 2010). Instead, in taking a developmental systems approach, the biological embodiment of infants and parents results in forms of engagement with each other. The question then is, what are the biological characteristics of human embodiment that make history and societies both possible and necessary (Elias, 1978)? Infants are born helpless and their embodiment, in contrast to chimpanzees and bonobos, results in social forms of interaction and thus in different developmental pathways. There is a lot more to be said here (see, e.g., Reddy, 2011, 2018; Savage-Rumbaugh and Fields, 2011; Dahl, 2017), but we will focus on forms of relations beginning at the end of the first year of life.

Rheingold (1982) insight regarding the importance of infants’ interest in participating in the activities of others has now been revived. This is the idea that infants’ early activities such as those labeled helping may initially not be motivated just by concern for others’ welfare but instead are more likely due to their interest in engaging with others in their activity. What are labeled prosocial behaviors in infants may not initially be motivated in terms of acting for others’ welfare. Instead, these activities may at first be motivated by interest and enjoyment in engaging in interaction with others (e.g., Carpendale et al., 2015; Dahl and Brownell, 2019; Dahl and Paulus, 2019; Carpendale and Lewis, 2021).

In taking this perspective, the question becomes how is it that infants develop what Rheingold (1982, p. 115) insightfully pointed out was characteristic of human infants which are “their interest in people and their activities”? We need to trace the developmental emergence of infants’ interest in engaging with other people. We briefly outline infants’ development in learning new ways to achieve engaging with adult’s attention—i.e., social interaction as a goal in itself—drawing on Bates and Reddy and others. In the context of communicative development within parent–infant interaction, Bates et al. (1975, p. 213) suggested that “the mutual joy taken in such interactions provides the first loop in the construction of declarative communication: the formulation of social interaction as a goal in itself.” Bates et al. (1975) further described the increasingly complex ways in which infants attempt to reach the goal of social interaction such as by engaging with others’ attention in various ways from showing objects, to showing off, to giving objects (Carpendale et al., 2021a; Broesch and Carpendale, 2022). Reddy (2003, 2011) also describes how infants engage with others’ attention such as through joking, humor, and clowning. This is not just a frivolous activity but reflects their interest and enjoyment of engaging with others.

In explicating the transitions in prosocial development, Dahl and Paulus (2019, p. 11) outline four phases in a sequence in early prosocial development in a pathway to altruism from “interest to obligation”: “(a) social preference for interacting with others (prealtruistic), (b) preference for action fulfillment (prealtruistic), (c) concern with promoting others’ well-being (altruistic), and (d) norm-based concerns (altruistic).” The second phase, to help achieve an action goal, is likely linked to learning about social routines and the ways actions are completed. This interest in others’ instrumental goals still does not necessarily require concern for others’ welfare, which is a later form in this development (Dahl and Paulus, 2019).

We have emphasized that development occurs within a system so it is important to consider the various factors involved such as the child’s characteristics as well as the influence of parents and the culture. For example, Newton et al. (2014) reported longitudinal bidirectional influences between maternal sensitivity and children’s prosocial behaviors over middle childhood.

## Relations among forms of prosocial action

Various forms of prosocial action have been studied. In particular, helping, sharing, and comforting have been focused on. An apparent puzzle that has emerged in this area of research is the finding that forms of prosocial behavior such as helping, sharing, and comforting tend not to be correlated in early development and seem to have different developmental trajectories (Laible and Karahuta, 2014; Paulus, 2014, 2018). The first step is to consider why that finding might be unexpected. If these are all examples of prosocial behavior that are motivated by concern for others, then once an infant can engage in one form, it might be expected that she would also engage in other forms. However, we refer to this as “an apparent puzzle” because it arises based on certain presuppositions, and not from other perspectives.

One approach to explaining this finding is that different forms of prosocial behavior involve different social-cognitive constraints (Dunfield, 2014). This follows from the assumption that it is social-cognitive abilities that make these relations possible. This is the idea that “early prosocial behaviors rest on the ability to recognize that another is having a negative experience, the ability to determine what an appropriate response would entail, and finally, the motivation to intervene” (Dunfield, 2014, p. 3). That is, the infant has to represent the problem, represent the solution, and have the motivation to carry out the action. From this perspective, the process is conceptualized as involving recognition and response to some negative state in others. Dunfield (2014, p. 3) suggests that “prosocial behaviors can be thought to require three components: (1) the ability to take the perspective of another person and recognize that they are having a problem; (2) the ability to determine the cause of that problem; and (3) the motivation to help them overcome the problem.” A general motivation to counteract the negative state of the other would be assumed, but different social cognitive skills may be required to engage in different forms of prosocial behavior. The various forms of prosocial behavior emerge at slightly different ages. Helping develops first during the first half of the second year, sharing later in the second year, and finally comforting (Dunfield, 2014).

However, this presupposes that all of these early activities are prosocial. But earlier we have reviewed objections to this assumption (e.g., Carpendale et al., 2015; Dahl and Paulus, 2019). Furthermore, we questioned the assumption that it is cognition that makes early relations possible. Instead, from an action-based perspective, we argue that various forms of prosocial behavior may emerge in different ways. The evidence of a lack of correlations among various forms of prosocial behavior in young children is a puzzle from a cognitivist/individualist perspective, but not, we suggest, from an action-based perspective. The cognitivist perspective begins from thinking and mind to get to relations, but from a relational perspective, this is backward and does not allow for an explanation of how thinking and mind emerge, instead they must just be given. By contrast, from a process-relational perspective, we begin from relations and cognition emerges from this. These forms of prosocial behavior all emerge within different activities. From this perspective, helping begins with children enjoying participating in the activities of adults (Bates et al., 1975; Rheingold, 1982; Reddy, 2003; Carpendale et al., 2015; Dahl and Paulus, 2019).

Most of the work on prosocial development from a process-relational perspective has focused on helping, but we now briefly extend this to consider sharing and comforting. Different developmental pathways likely underlie the different forms of prosocial behavior. Sharing may be linked to giving and may emerge within routines at least partially and initially initiated by parents (Carpendale et al., 2021a; Wallbridge, 2022). Some early examples of what looks like sharing might be explained in other ways. For example, a child at 21 months upon opening an Easter egg and seeing three chocolates immediately gave one to her mother, one to her father, and kept one for herself (Carpendale et al., 2021a). But what happened later that day with Easter eggs and chocolates suggest that this child still had much to learn about sharing. The fact that that this initial interaction resulted in equality might be due to seeing the matching of three objects and three people. Progress through the various forms of interaction leading to sharing may be driven by young children learning about the social and emotional consequences of their actions for themselves as well as the others whom they are interacting with. For example, preschoolers’ inclination to share resources with others can be facilitated by their anticipation of others’ potential negative emotions if they are not shared with (Paulus and Moore, 2015). Furthermore, preschool-aged children do understand that sharing with others does make one happy, and children who are more aware of their potential for feeling good after sharing tended to share more than other children (Paulus and Moore, 2016). Through such experiences, children develop an understanding of such social situations by coming to anticipate the positive and negative emotional consequences for all involved—self and others.

Comforting is yet another complex prosocial activity that may have had the least attention from an action-based perspective. It can be conceptualized from the cognition-first perspective based on theories about empathy such as Martin Hoffman’s theory (Johansson, 2008). By contrast, it can be approached from a relations-first perspective in which the focus is on the world that infants are immersed in and learn the typical patterns of interaction (Johansson, 2008). This social skill of comforting needs further investigation with naturalistic observations, but here are some examples. An infant at 10 months while nursing saw his mother massage his father’s sore back. He stopped nursing, sat up, and started rubbing his father’s back. His mother reported that this “was so sweet” (Carpendale et al., 2015). Thus, his action was likely responded to positively by her and was an enjoyable experience for the child. This could be considered similar to helping because he was participating in his mother’s activity, or it could be considered comforting, but it is very unlikely that the infant at 10 months understood his action as comforting. In an example with an older child at 2 years and 6 months, she saw her mother lying on the couch and not feeling well. The child’s response was, “do not worry mama, I’ll help you feel better. How about a toy?” And she found a toy and gave it to her mother (Carpendale et al., 2021a). This interaction involves giving and sharing and can also be considered comforting. The difference between these two forms of interaction at 10 months and at 30 months shows the gap that must be filled in with a developmental explanation.

Comforting others in older children involves awareness of others’ distress and concern for them, thus there is an emotional underpinning in this development. We should also consider the role of emotions in all forms of prosocial action, although they may be different emotions. In the context of sharing, we note the influence of anticipating the positive and negative consequences for self and others of sharing or not sharing. Regarding helping, interest may initially draw children into the activity, and concern for others may become more important later in development. In the context of developing skills in comforting others, an important emotional context is that infants are responsive to the distress of others. But it is not clear if this empathic reaction initially reflects an understanding of the emotional distress of others and a concern for others (e.g., Laible and Karahuta, 2014). Instead, this is something that must develop. This early empathic response is a form of coordination with others at a pre-reflective level of interactivity. In this early form of participating in social interactivity, we can see the emergence of perspectives at a pre-reflective level. This is an indication of the origin of perspectives in our relations with others and the world that we live in, and in taking this approach, we begin with interactivity as primarily social and relational rather than cognitive, mentalistic, and individual (Martin et al., 2008). This is the context in which infants learn about the self and others. Further development toward reflective interactivity is facilitated by language development and becoming able to talk about emotions in oneself and others and thus to become aware of and able to reflect on such emotions. Learning to talk about emotions is based on infants’ experience in interaction with others in which emotions are expressed and talked about (e.g., Carpendale and Lewis, 2021). This skill in the use of language about emotions results in the further development of a concern for others.

The two observations provide examples at varying ages and levels of complexity and illustrate how they are based on social routines the child is learning about in their homes with their caregivers. It is also likely that caregivers appreciate such activities and that it results in an enjoyable interaction for both caregivers and children and that this would scaffold the development of such skills (Dahl et al., 2011). These activities emerge and develop within relations and so this involves learning routines with parents. But characteristics of children such as their sociability will also influence the interaction they elicit and in which they develop (Paulus, 2018). In further developments, these various prosocial activities may likely become increasingly correlated, for example, children may begin to see these activities as linked to being a good and nice person and thus linked to their sense of self as a good person (Paulus, 2018).

Here, we have far too briefly touched on a complex theoretical and empirical issue regarding the interrelations among forms of prosocial action, but we want to suggest ways in which this issue could be grappled with from an action-based, process-relational perspective, in which rather than beginning with cognition to explain relations, we begin with relations to explain the development of cognition. We argue that increasingly complex forms of prosocial behavior are not primarily explained by more complex social cognitive abilities, but rather that children acquire more experience in typical social routines and can anticipate outcomes of interaction so that it is their emerging skill within these relations that explains the development of social cognitive abilities.

## Considering examples of research that might clash with a process-relational perspective

We would now like to further clarify our position by considering examples of research on prosocial development that might at first seem to be inconsistent with our approach. Because we have emphasized development within social contexts, we might overlook neurological development. For example, distinct patterns of neural activity are linked to different forms of prosocial action (Paulus et al., 2013). Although this finding might appear to be inconsistent with our approach, it is expected from our perspective because, consistent with neuroconstructivism, we assume that activity shapes neural pathways (Mareschal et al., 2007). Stiles and her colleagues further explicate the neuronal processes involved in the strengthening of synapses. Thus, particular forms of neural activity would be expected to be linked to particular social activities (Mareschal et al., 2007; Stiles, 2009; Stiles et al., 2015).

A line of research drawn on to support claims that prosociality is “rooted deeply in human nature” (Hepach, 2017, p. 50) makes use of measuring infants’ pupil dilation in an attempt to measure their emotional processes. Hepach et al. (2012) measured 2-year-olds’ pupil dilation and found no difference between the conditions in which a child helped the adult needing help or a third person provided the help rather than the child. They interpret this as supporting their social arousal hypothesis and the claim that children are intrinsically altruistic and just want to see the person helped but do not need to perform the action themselves. This might seem to be inconsistent with our view that early forms of infants’ helping might be due to their interest in being involved in the activities of adults. A problem with this methodology, however, is that pupil dilation is not specific, and it might be the result of various cognitive and emotional processes. It might indicate “increased attention, emotional arousal, cognitive effort such as memory processes, target detection and/or surprise” (Pletti et al., 2017, p. 2). Although Hepach et al. (2012) assert that their results are consistent with their hypothesis, they have not ruled out other possible hypotheses. The third condition in their experiment in which they did find increased pupil dilation was when the child was prevented from helping the adult. Increased arousal in this condition is consistent with the other hypotheses that the child was interested in engaging in interaction, wished to fulfill the adult’s goal, or wanted to complete the task, or it could be due to greater memory load in that condition (Pletti et al., 2017).

Hepach (2017) claims that prosociality is “rooted deeply in human nature” (p. 50). Whether we agree with this claim depends crucially on what is meant. If he means that prosocial action is a form of activity that tends to reliably emerge within human developmental systems, then this would likely receive the broad agreement. But it is still necessary to explain how it develops, and in doing so, we take a developmental systems perspective. However, we suspect that Hepach is making a stronger claim and is suggesting that this is somehow “biological.” But this assumes a split between biological and social processes, and it is not clear what biological processes can get him from the zygote to intrinsic altruism, and he does not cite any biological sources. If we read the work of geneticists and neuroscientists, it seems that they are not happy with the strong claims made by psychologists. Instead, we have to describe the developmental processes involved (e.g., Fisher, 2006; Gottlieb, 2007; Stiles, 2009; Meaney, 2010).

There are also other claims that we suggest are based on problematic methodologies. For example, Köster et al. (2016) interpret their results as indicating that 9-to 18-month-old infants understand others’ needs in a helping situation. We are cautious about this interpretation, given the method, it is based on. Paulus (2022) argued that the VoE method is based on questionable assumptions and is too speculative to be relied on (see also Tafreshi et al., 2014). From our action-based perspective, infants are gradually learning about others’ needs and goals in increasingly complex situations through learning to anticipate outcomes of actions. Thus, their understanding of goals at this point is at the sensorimotor level, and we resist assuming a mentalistic perspective. It does not seem likely that understanding the situation in this study is already mastered at 9 months. Understanding others’ needs will certainly become an important factor in explaining later prosocial actions such as helping and comforting. But as developmentalists, is it our job to explain the development of such skills rather than presuppose them. This is our goal in taking a process-relational, action-based, developmental systems approach.

To be clear, we suggest that toddlers’ beginning engagement in activities that we refer to as helping may be due to their interest in participating in the activities of adults, a point made by Rheingold (1982). However, through continued experience in such activities, children will learn about the social contexts and the positive and negative emotional consequences of such activities for the self and others, and thus their reasons for engaging in such actions will change over development. Furthermore, in the context of other activities, considered prosocial, such as sharing and comforting, other factors, such as empathic concern regarding others’ distress, may be important in initially drawing infants into these patterns of interaction that may differ from those that draw them into helping.

## Methodology following from a process-relational perspective

We have argued that the presuppositions our theories are based on influence how we frame questions and draw conclusions. They also influence the form of the methodology used in research. If it is correct that children learn to act to benefit others within their relations with others, then it follows that a way to study this development is to observe it as it is in the process of emergence within the interaction. This is a methodology that follows from the perspective we take, and it requires detailed longitudinal naturalistic observations. Psychology, however, has been critiqued as being observation-and description-deprived (ODD; Rai and Alan Fiske, 2010; see also Dahl, 2017). From our perspective, naturalistic observations are essential in studying development, and a detailed longitudinal description of the emergence of a skill is a developmental explanation for the development of such skills (Hendriks-Jansen, 1996).

There are several ways in which to obtain such detailed longitudinal observations. Ideally, the researcher could always be present to make such observations, but this is rarely the case unless the researcher is also the caregiver. Alternatives are recording observations either with video at regular, and hopefully closely spaced, visits or with parental diary observations used to fill in gaps in development. Diary studies can be conducted as case studies or multiple case studies (Carpendale and Carpendale, 2010; Kettner and Carpendale, 2018). It is also possible to combine both designs to obtain parental observations during the time between home visits because otherwise crucial transitional phenomena might be missed (Wallbridge, 2022). Infant development is variable; either significant changes can occur over a short time or there can be little change. Good observations require recording enough detail and this means that talented and interested observers are needed. Researchers can provide some training, but parents do need to be interested and willing to devote the time required.

Based on expectation that social skills emerge within interaction, we suggest close observation with dyads or families and multiple case studies following dyads or families; the emphasis would be on more, and more detailed, observations rather than more participants. It is also important to be cautious about averaging across many participants in case important differences in developmental pathways are obscured. Differences in developmental pathways can be due to infants’ differences as well as parental and cultural differences.

## Conclusion

We have argued that the presuppositions researchers begin with already structure the possible answers to their questions, as well as the methods they draw on so that the philosophical assumptions they take for granted follow them through the design of their empirical work to their theoretical conclusions. Thus, we have suggested that it behooves us to examine these crucial assumptions. We have critiqued the cognitivist perspective, according to which it is necessary to begin from cognition to explain human relations. We have suggested an alternative worldview according to which it is relations that are primary, and these relations account for the development of human forms of thinking, rather than the other way around. Furthermore, these relations are ethical in their nature (Jopling, 1993). We have applied this framework—sometimes referred to as a process-relational worldview—to account for the development of prosocial action. An ethical dimension is already there in responsiveness because we treat others as someone, not something (Spaemann, 2006), and this is Buber (1958) distinction between “I-thou” versus “I-it.” We suggest that this is a fruitful perspective from which to view the emergence of prosocial and moral development. In this article, we have focused on early prosocial development and have discussed possible pathways in the development of various forms of prosocial action such as helping, sharing, and comforting. Accounting for further moral development and the emergence of views about what is right and wrong involves additional development, which we suggest involves arriving at an interpersonal agreement through coordinating conflicting perspectives (see Carpendale et al., 2013, 2021a). In addition, we have briefly sketched the methodology that follows from this metatheoretical perspective.

## Author contributions

JC wrote the first draft. BW contributed sections to the manuscript. Both authors contributed to the article and approved the submitted version.

## Funding

This study was funded by the Simon Fraser University.

## Conflict of interest

The authors declare that the research was conducted in the absence of any commercial or financial relationships that could be construed as a potential conflict of interest.

## Publisher’s note

All claims expressed in this article are solely those of the authors and do not necessarily represent those of their affiliated organizations, or those of the publisher, the editors and the reviewers. Any product that may be evaluated in this article, or claim that may be made by its manufacturer, is not guaranteed or endorsed by the publisher.

## References

[ref1] Allen J. Bickhard M. (2013). Stepping off the pendulum: why only an action-based approach can transcend the nativist–empiricist debate. Cogn. Dev. 28, 96–133. doi: 10.1016/j.cogdev.2013.01.002

[ref2] Bates E. Camaioni L. Volterra V. (1975). The acquisition of performatives prior to speech. Merrill-Palmer Q. 21, 205–226.

[ref500] Bian L. Sloane S. Baillargeon R. (2018). Infants expect ingroup support to override fairness when resources are limited. Proc. Natl. Acad. Sci. 115, 2705–2710. doi: 10.1073/pnas.1719445115, PMID: 29483252 PMC5856544

[ref3] Bibok M. B. Carpendale J. I. M. Lewis C. (2008). “Social knowledge as social skill: an action based view of social understanding” in Social life and social knowledge: Toward a process account of development. eds. Müller U. Carpendale J. I. M. Budwig N. Sokol B. (New York: Taylor Francis), 145–169.

[ref4] Bickhard M. (2009). The interactivist model. Synthese 166, 547–591. doi: 10.1007/s11229-008-9375-x

[ref5] Bloom P. (2010). The moral life of babies. New York: New York Times.

[ref6] Bloom P. (2012). “Moral nativism and moral psychology” in The social psychology of morality: Exploring the causes of good and evil. eds. Mikulincer M. Shaver P. R. (Washington: American Psychological Association), 71–89. doi: 10.1037/13091-004

[ref7] Broesch T. Carpendale J. I. M. (2022). “Emotional development across cultures” in Oxford handbook of emotional development. eds. Dukes D. Samson A. C. Walle E. A. (New York: Oxford University Press), 398–406.

[ref8] Buber M. (1958). I and thou (2nd ed.). New York: Charles Scribner’s Sons.

[ref9] Buyukozer Dawkins M. B. Sloane S. Baillargeon R. (2019). Do infants in the first year of life expect equal resource allocations? Front. Psychol. 10:116. doi: 10.3389/fpsyg.2019.00116, PMID: 30837906 PMC6389704

[ref10] Carpendale J. I. M. (2018). “Communication as the coordination of activity: the implications of philosophical preconceptions for theories of the development of communication” in Advancing developmental science: Philosophy, theory, and method. eds. Dick A. S. Müller U. (New York: Routledge, Taylor and Francis group), 145–156.

[ref11] Carpendale J. I. M. (2020). “The developmental roots of respect and its role in development” in Peer commentary on the article “the development of respect in children and adolescents”. eds. Malti T. Peplak J. Zhang L. (Monograph Matters) https://monographmatters.srcd.org/2020/08/11/commentary-carpendale-85-3/

[ref12] Carpendale J. I. M. Carpendale A. B. (2010). The development of pointing: from personal directedness to interpersonal direction. Hum. Dev. 53, 110–126. doi: 10.1159/000315168

[ref14] Carpendale J. I. M. Hammond S. I. Atwood S. (2013). “A relational developmental systems approach to moral development” in Embodiment and epigenesis: Theoretical and methodological issues in understanding the role of biology within the relational developmental system. eds. Lerner R. M. Benson J. B., vol. 45 (San Diego, CA: Advances in Child Development and Behavior), 105–133.

[ref15] Carpendale J. I. M. Kettner V. A. Audet K. N. (2015). On the nature of toddlers’ helping: helping or interest in others’ activity? Soc. Dev. 24, 357–366. doi: 10.1111/sode.12094

[ref16] Carpendale J. I. M. Lewis C. (2020). Tomasello’s tin man of moral obligation needs a heart. Behav. Brain Sci. 43, 19–20. doi: 10.1017/S0140525X19002462 32349811

[ref17] Carpendale J. I. M. Lewis C. (2021). What makes us human? How minds are constructed in relationships. New York: Routledge.

[ref18] Carpendale J. I. M. Müller U. Wallbridge B. Broesch T. Cameron-Faulkner T. Ten Eycke K. (2021a). The development of giving in forms of object-exchange: exploring the roots of communication and morality in early interaction around objects. Hum. Dev. 65, 166–179. doi: 10.1159/000517221

[ref19] Carpendale J. I. M. Müller U. Wallbridge B. Lewis C. (2021b). “From neurons to knowing: implications of theoretical approaches for conceptualizing and studying the neural bases of social understanding” in The neural bases of mentalizing. eds. Ochsner K. Gilead M. (Cham, Switzerland: Springer Press), 317–334.

[ref20] Carpendale J. I. M. Parnell V. L. Wallbridge B. (2021c). Conceptualizations of knowledge in structuring approaches to moral development: a process-relational approach. Front. Psychol. 12:756654. doi: 10.3389/fpsyg.2021.756654, PMID: 34975648 PMC8716751

[ref21] Chomsky N. (2007). Biolinguistic explorations: design, development, evolution. Int. J. Philos. Stud. 15, 1–21. doi: 10.1080/09672550601143078

[ref22] Dahl A. (2017). Ecological commitments: why developmental science needs naturalistic methods. Child Dev. Perspect. 11, 79–84. doi: 10.1111/cdep.12217, PMID: 28584562 PMC5455774

[ref23] Dahl A. Brownell C. (2019). The social origins of human prosociality. Curr. Dir. Psychol. Sci. 28, 274–279. doi: 10.1177/0963721419830386, PMID: 31602098 PMC6786781

[ref24] Dahl A. Campos J. J. Witherington D. C. (2011). Emotional action and communication in early moral development. Emot. Rev. 3, 147–157. doi: 10.1177/1754073910387948

[ref26] Dahl A. Paulus M. (2019). From interest to obligation: the gradual development of human altruism. Child Dev. Perspect. 13, 10–14. doi: 10.1111/cdep.12298

[ref27] De Jaegher H. Di Palolo E. Gallagher S. (2010). Can social interaction constitute social cognition? Trends Cogn. Sci. 14, 441–447. doi: 10.1016/j.tics.2010.06.009 20674467

[ref28] De Jaegher H. Palolo D. (2007). Participatory sense-making: an enactive approach to social cognition. Phenomenol. Cogn. Sci. 6, 485–507. doi: 10.1007/s11097-007-9076-9

[ref29] Dunfield K. A. (2014). A construct divided: prosocial behavior as helping, sharing, and comforting subtypes. Front. Psychol. 5:958. doi: 10.3389/fpsyg.2014.00958, PMID: 25228893 PMC4151454

[ref30] Elias N. (1978). What is sociology? New York: Columbia University Press.

[ref31] Fisher S. E. (2006). Tangled webs: tracing the connections between genes and cognition. Cognition 101, 270–297. doi: 10.1016/j.cognition.2006.04.004, PMID: 16764847

[ref32] Fuchs T. (2013). The phenomenology and development of social perspectives. Phenomenol. Cogn. Sci. 12, 655–683. doi: 10.1007/s11097-012-9267-x

[ref33] Gilligan C. (1982). In a difference voice. Cambridge, MA: Harvard University Press.

[ref35] Gottlieb G. (2007). Probablistic epigenesis. Dev. Sci. 10, 1–11. doi: 10.1111/j.1467-7687.2007.00556.x 17181692

[ref36] Graham J. Haidt J. Koleva S. Motyl M. Iyer R. Wojcik S.. (2013). Moral foundations theory: the pragmatic validity of moral pluralism. Adv. Exp. Soc. Psychol. 47, 55–130. doi: 10.1016/B978-0-12-407236-7.00002-4

[ref37] Griffiths P. E. Stotz K. (2000). How the mind grows: a developmental perspective on the biology of cognition. Synthese 122, 29–51. doi: 10.1023/A:1005215909498

[ref38] Hamlin J. K. (2013). Moral judgment and action in preverbal infants and toddlers: evidence for an innate moral core. Curr. Dir. Psychol. Sci. 22, 186–193. doi: 10.1177/0963721412470687

[ref39] Hamlin J. K. Wynn K. Bloom P. (2007). Social evaluation by preverbal infants. Nature 450, 557–559. doi: 10.1038/nature06288 18033298

[ref40] Hammond S. Drummond J. K. (2019). Rethinking emotions in the context of infants’ prosocial behavior: the role of interest and positive emotions. Dev. Psychol. 55, 1882–1888. doi: 10.1037/dev0000685, PMID: 31464492

[ref41] Hauser M. D. (2006a). Moral minds: How nature designed our universal sense of right and wrong. New York: HarperCollins.

[ref42] Hauser M. D. (2006b). The liver and the moral organ. Scan 1, 214–220. doi: 10.1093/scan/nsl026, PMID: 18985108 PMC2555424

[ref43] Heil J. (1981). Does cognitive psychology rest on a mistake? Mind XC, 321–342. doi: 10.1093/mind/XC.359.321

[ref44] Hendriks-Jansen H. (1996). Catching ourselves in the act. Cambridge, MA: The MIT Press, doi: 10.7551/mitpress/1748.001.0001.

[ref45] Hepach R. (2017). Prosocial arousal in children. Child Dev. Perspect. 11, 50–55. doi: 10.1111/cdep.12209

[ref46] Hepach R. Vaish A. Tomasello M. (2012). Young children are intrinsically motivated to see others helped. Psychol. Sci. 23, 967–972. doi: 10.1177/0956797612440571, PMID: 22851443

[ref47] Hobson R. P. (2004). The cradle of thought. London: Macmillan/Oxford University Press.

[ref48] Hobson R. P. (2012). Emotion as personal relatedness. Emot. Rev. 4, 169–175. doi: 10.1177/1754073911430141

[ref49] Hutto D. D. Myin E. (2013). Radicalizing enactivism: Basic minds without content. Cambridge, MA: The MIT Press.

[ref50] Johansson E. (2008). Empathy or intersubjectivity? Understanding the origins of morality in young children. Stud. Philos. Educ. 27, 33–47. doi: 10.1007/s11217-007-9046-2

[ref51] Jopling D. (1993). “Cognitive science, other minds, and the philosophy of dialogue” in The perceived self. ed. Neisser U. (Cambridge, MA: MIT Press), 290–309.

[ref52] Kettner V. Carpendale J. I. M. (2018). From touching to communicating: forms of index finger use in the development of pointing. Gesture 17, 245–267. doi: 10.1075/gest.18005.ket

[ref55] Köster M. Ohmer X. Nguyen T. D. Kärtner J. (2016). Infants understand others’ needs. Psychol. Sci. 27, 542–548. doi: 10.1177/0956797615627426 26902106

[ref56] Laible D. Karahuta E. (2014). “Prosocial behaviors in early childhood: helping others, responding to the distress of others, and working with others” in Prosocial development: A multidimensional approach. eds. Padilla-Walker L. M. Carlo G. (Oxford: Oxford University Press), 350–373. doi: 10.1093/acprof:oso/9780199964772.003.0017

[ref57] Lerner R. M. (2016). Complexity embraced and complexity reduced: a tale of two approaches to human development. Hum. Dev. 59, 242–249. doi: 10.1159/000452113

[ref59] Mareschal D. Johnson M. H. Sirois S. Spratling M. W. Thomas M. S. C. Westermann G., (2007). Neuroconstructivism: How the brain constructs cognition (vol. 1). New York: Oxford University Press, doi: 10.1093/acprof:oso/9780198529910.001.0001. 18578929

[ref60] Margolis E. Laurence S. (2013). In defense of nativism. Philos. Stud. 165, 693–718. doi: 10.1007/s11098-012-9972-x

[ref61] Martin J. Sokol B. W. Elfers T. (2008). Taking and coordinating perspective: from prereflective interactivity, through reflective intersubjectivity, to metareflective sociality. Hum. Dev. 51, 294–317. doi: 10.1159/000170892

[ref62] Maturana H. R. Varela F. J. (1987). The tree of knowledge: The biological roots of human understanding. Boston, Massachusetts: Shambhala Publications, Inc.

[ref64] Mead G. H. (1934). Mind, self and society: From the standpoint of a social behaviorist. Chicago: University of Chicago Press.

[ref65] Meaney M. J. (2010). Epigenetics and the biological definition of gene x environment interactions. Child Dev. 81, 41–79. doi: 10.1111/j.1467-8624.2009.01381.x 20331654

[ref66] Mikhail J. (2007). Universal moral grammar: theory, evidence and the future. Trends Cogn. Sci. 11, 143–152. doi: 10.1016/j.tics.2006.12.007, PMID: 17329147

[ref67] Newen A. De Bruin L. Gallagher S. (Eds.), (2018). The Oxford handbook of 4E cognition. Oxford, UK: Oxford University Press, doi: 10.1093/oxfordhb/9780198735410.001.0001.

[ref68] Newen A. Gallagher S. De Bruin L. (2018). 4D cognition: historical roots, key concepts, and central issues in Newen A. Bruin L. De Gallagher S., (Eds.), The Oxford handbook of 4E cognition, 2–16. Oxford, UK: Oxford University Press. doi:10.1093/oxfordhb/9780198735410.013.1

[ref69] Newton E. K. Laible D. Carlo G. Steele J. S. McGinley M. (2014). Do sensitive parents foster kind children, or vice versa? Bidirectional influences between children’s prosocial behavior and parental sensitivity. Dev. Psychol. 50, 1808–1816. doi: 10.1037/a0036495, PMID: 24708456

[ref70] Noddings N. (1984). Caring: A feminine approach to ethics and moral education. Berkeley: University of California Press.

[ref71] Overton W. F. (2015). “Processes, relations, and relational-developmental-systems theory and method” in Handbook of child psychology and developmental science. eds. Overton W. F. Molenaar P. C. M. Lerner R., vol. 1. 7th ed (Oxford: John Wiley & Sons, Inc.), 9–62. doi: 10.1002/9781118963418.childpsy102

[ref72] Paulus M. (2014). The emergence of prosocial behavior: why do infants and toddlers help, comfort, and share? Child Dev. Perspect. 8, 77–81. doi: 10.1111/cdep.12066

[ref73] Paulus M. (2018). The multidimensional nature of early prosocial behavior: a motivational perspective. Curr. Opin. Psychol. 20, 111–116. doi: 10.1016/j.copsyc.2017.09.003, PMID: 28988024

[ref74] Paulus M. (2022). Should infant psychology rely on the violation-of-expectation method? Not anymore. Infant Child Dev. 31:2306. doi: 10.1002/icd.2306

[ref75] Paulus M. Kühn-Popp N. Licata M. Sodian B. Meinhardt J. (2013). Neural correlates of prosocial behavior in infancy: different neurophysiological mechanisms support the emergence of helping and comforting. Neuro Image 66, 522–530. doi: 10.1016/j.neuroimage.2012.10.041, PMID: 23108275

[ref76] Paulus M. Moore C. (2015). Preschool children’s anticipation of recipients’ emotions affects their resource allocation. Soc. Dev. 24, 852–867. doi: 10.1111/sode.12126

[ref77] Paulus M. Moore C. (2016). Preschoolers’ generosity increases with understanding of the affective benefits of sharing. Dev. Sci. 20:e12417. doi: 10.1111/desc.12417, PMID: 27256161

[ref78] Piaget J. (1965). The moral judgment of the child. New York: The Free Press.

[ref79] Pinker S. (1994). The language instinct: How the mind creates language. New York: Harper Perennial.

[ref80] Pletti C. Scheel A. Paulus M. (2017). Intrinsic altruism or social motivation–what does pupil dilation tell us about children's helping behavior? Front. Psychol. 8:2089. doi: 10.3389/fpsyg.2017.02089, PMID: 29259566 PMC5723414

[ref81] Portmann A. (1990). A zoologist looks at humankind. New York: Columbia University Press.

[ref82] Rai T. S. Alan Fiske A. (2010). ODD (observation-and description-deprived) psychological research. Behav. Brain Sci. 33, 106–107. doi: 10.1017/S0140525X10000221, PMID: 20546657

[ref83] Reddy V. (2003). On being the object of attention: implications for self-other consciousness. Trends Cogn. Sci. 7, 397–402. doi: 10.1016/S1364-6613(03)00191-8, PMID: 12963470

[ref84] Reddy V. (2011). “A gaze at grips with me” in Joint attention: New developments in psychology, philosophy of mind, and social neuroscience. ed. Seemann A. (Cambridge, MA: MIT Press), 137–157.

[ref85] Reddy V. (2018). Why engagement? A second-person take on social cognition in Newen A. Bruin L. de Gallagher S. (Eds.), The Oxford handbook of 4E cognition, 433–452. Oxford, UK: Oxford University Press.

[ref86] Reddy V. Vanello D. (2022). “Emotional engagement and social understanding in early development” in The Oxford handbook of emotional development. eds. Dukes D. Samson A. C. Walle E. A. (Oxford, UK: Oxford University Press), 249–260.

[ref87] Rheingold H. L. (1982). Little children’s participation in the work of adults, a nascent prosocial behavior. Child Dev. 53, 114–125. doi: 10.2307/1129643

[ref88] Ross R. (2006). Returning to the teachings: Exploring aboriginal justice. Canada: Penguin.

[ref89] Savage-Rumbaugh E. S. Fields W. M. (2011). “The evolution and the rise of human language: carry the baby” in Homo symbolicus: The dawn of language, imagination and spirituality. eds. Henshilwood C. S. d’Errico F. (Amsterdam: John Benjamins Publishing Company), 13–48. doi: 10.1075/z.168.02sav

[ref90] Spaemann R. (2006). Persons: The difference between ‘someone’ and ‘something’. Oxford: Oxford University Press original work published 1996).

[ref91] Stiles J. (2009). On genes, brains, and behavior: why should developmental psychologists care about brain development? Child Dev. Perspect. 3, 196–202. doi: 10.1111/j.1750-8606.2009.00106.x

[ref92] Stiles J. Brown T. T. Haist F. Jernigan T. L. (2015). “Brain and cognitive development cognitive processes” in Handbook of child psychology and developmental science. eds. Liben L. Müller U. Lerner R., vol. 2. 7th ed (New York: John Wiley & Sons, Inc.), 1–54. doi: 10.1002/9781118963418.childpsy202

[ref93] Tafreshi D. Thompson J. J. Racine T. P. (2014). An analysis of the conceptual foundations of the infant preferential looking paradigm. Hum. Dev. 57, 222–240. doi: 10.1159/000363487

[ref95] Tomasello M. (2020). The moral psychology of obligation. Behav. Brain Sci. 43, 1–58. doi: 10.1017/S0140525X19001742 31133086

[ref96] Tutu D. (1999). No future without forgiveness. New York Image 16, 29–30. doi: 10.1111/j.1540-5842.1999.tb00012.x

[ref97] Waghid Y. Smeyer P. (2012). Reconsidering Ubuntu: on the educational potential of a particular ethic of care. Educ. Philos. Theory 44, 6–20. doi: 10.1111/j.1469-5812.2011.00792.x

[ref98] Wallbridge J. B. (2022). The development of object extension gestures: An action-based approach. Canada: Simon Fraser University.

[ref99] Warneken F. (2016). Insights into the biological foundation of human altruistic sentiments. Curr. Opin. Psychol. 7, 51–56. doi: 10.1016/j.copsyc.2015.07.013

[ref101] Wittgenstein L. (2009). Philosophical investigations. (revised 4th ed., translation by Anscombe G.E.M. Hacker P.M.S. Schulte J.). New York: Wiley-Blackwell. original work published 1953

